# The effects of red LED light on pig sperm function rely upon mitochondrial electron chain activity rather than on a PKC-mediated mechanism

**DOI:** 10.3389/fcell.2022.930855

**Published:** 2022-10-07

**Authors:** Olga Blanco-Prieto, Carolina Maside, Àlex Peña, Júlia Ibáñez-Príncep, Sergi Bonet, Marc Yeste, Joan E. Rodríguez-Gil

**Affiliations:** ^1^ Department of Animal Medicine and Surgery, Faculty of Veterinary Medicine, Autonomous University of Barcelona, Barcelona, Spain; ^2^ Department of Veterinary Medicine Science, University of Bologna, Bologna, Italy; ^3^ Biotechnology of Animal and Human Reproduction (TechnoSperm), Institute of Food and Agricultural Technology, University of Girona, Girona, Spain; ^4^ Unit of Cell Biology, Department of Biology, Faculty of Sciences, University of Girona, Girona, Spain; ^5^ Catalan Institution for Research and Advanced Studies (ICREA), Barcelona, Spain

**Keywords:** sperm, mitochondria, antimycin A, PKC, light irradiation

## Abstract

While irradiation with red LED light has been reported to modulate sperm function in different mammalian species, the mechanisms underlying their response are poorly understood. This work sought to provide new insights into whether this effect relies on a direct action upon mitochondrial electron chain and/or on PKC-linked mechanisms such as those related to opsins. For this purpose, pig semen was light-stimulated for 1, 5 or 10 min in the presence/absence of antimycin A, an inhibitor of the mitochondrial electron chain, or PKC 20–28^®^ (PKCi), a PKC inhibitor. Antimycin A completely blocked the effects of light at all the performed irradiation patterns. This effect was linked to a complete immobility of sperm, which was accompanied with a significant (*p* < 0.05) drop in several markers of mitochondrial activity, such as JC-1 staining and O_2_ consumption rate. Antimycin A, however, did not affect intracellular ATP levels, intramitochondrial calcium, total ROS, superoxides or cytochrome C oxidase (CCO) activity. In the case of PKCi, it did also counteract the effects of light on motility, O_2_ consumption rate and CCO activity, but not to the same extent than that observed for antimycin A. Finally, the effects observed when sperm were co-incubated with antimycin A and PKCi were similar to those observed with antimycin A alone. In conclusion, red LED light acts on sperm function via a direct effect on mitochondrial electron chain. Additionally, light-activated PKC pathways have a supplementary effect to that observed in the electron chain, thereby modulating sperm parameters such as motility and CCO activity.

## Introduction

In the last years, there has been increasing evidence about the effects of red light irradiation on sperm function, including *in vivo* fertilizing ability, in species from separate phyla such as birds, fishes and mammals (see Yeste et al., 2018 for review). Although, under a practical point of view, getting new insights into how light exerts the aforementioned effects could improve *in vivo* reproductive efficiency, the mechanisms underlying that response remain largely unknown. In addition, previous research has demonstrated that the sperm response to light depends on other factors, including the species, nature of semen (i.e., fresh, frozen-thawed), wavelength range and color of the recipient/straw (Catalán et al., 2020a; Catalán et al., 2020b; Blanco-Prieto et al., 2020; Catalán et al., 2020c; Catalán et al., 2020d; Catalán et al., 2021). In the case of pigs, different studies reported inconsistent results, as whereas Luther et al. (2019) found an impairment of sperm function, others observed a positive impact on sperm quality and fertilizing ability (Yeste et al., 2016; Blanco-Prieto et al., 2019; Pezo et al., 2019).

Thus far, irradiating sperm with a wavelength ranging from 610 nm to 640 nm (which corresponds to orange-to-red light) has the most positive effects (see Rodríguez-Gil, 2019 for review). The mechanisms through which red light could exert these outcomes have been suggested to combine different pathways that would act simultaneously. On this respect, there are at least three separate mechanisms that could explain light effects. The first mechanism would be related with a direct action of light on separate cell structures through the modification of covalent bonds. In effect, red light irradiation has been reported to induce an increase in the energy stored in phosphodiester bonds present in molecules such as ATP, thus increasing the energy stored by cells without modifying the total number of synthesized ATP molecules (Deng et al., 2020). While this direct effect is not well understood, the light-induced additional energy stored in the covalent bonds of a great number of molecules in addition to ATP should have an impact on the overall cell function. In any case, more in-depth studies are needed to get further insights into this mechanism.

The second mechanism would be related to a direct action on mitochondrial function, particularly, on different locations of the electron chain. Indeed, both cytochrome C and cytochrome P450 complexes are sensitive to light at 450 nm and 610–630 nm/660–680 nm, respectively (Lynch and Copeland, 1992). Furthermore, it has been described that red LED light has a direct effect on the mitochondrial electron chain of mammalian sperm through the modulation of cytochrome C oxidase (CCO) activity (Blanco-Prieto et al., 2020; Catalán et al., 2020d). Not only could the light-induced impact on sperm mitochondria affect their energy levels, but also other important cell events such as those related to the apoptotic-like changes occurring during sperm capacitation (Aitken et al., 2015). This effect could contribute to explain why irradiating sperm with red light can increase their fertilizing ability, despite this impact not being observed in all farms (Yeste et al., 2016; Blanco-Prieto et al., 2019).

Finally, a third mechanism would be related to the effect of red light on transmembrane proteins, particularly on ion channels pertaining to the Transient Receptor Potential (TRP) family (Vriens et al., 2014). The TRP family includes a fairly big, heterogeneous number of transmembrane proteins that are involved in the control of thermotaxis in eukaryotic cells (Vriens et al., 2014). In mammalian sperm, the TRPV4 vanilloid type of TRP receptor has been identified as the most important temperature-sensitive ion channel (Mundt et al., 2018). This is of interest, because thermotaxis has been described as a very important mechanism in the modulation of mammalian sperm function inside the female genital tract (Lottero-Leconte et al., 2017). Additionally, considering the extreme sensitivity of mammalian sperm to temperature changes as small as 0.0006°C (Hunter, 2012), a tight control of thermotaxis-modulating systems such as TRPV4 could be important for the regulation of sperm function after ejaculation. In fact, the relevance of thermotaxis is also evidenced by the fact that, apart from TRPs, there are other transmembrane temperature-sensitive proteins, such as opsins, that are related to this mechanism (Pérez-Cerezales et al., 2015; Rodríguez-Gil, 2019). One of the most conspicuous opsins detected in mammalian sperm is rhodopsin, which reaches its maximal sensitivity when stimulated by red light (Foster et al., 1991). For this reason, it could be hypothesized that the action of red LED light on sperm rhodopsin could be related to thermotaxis. In addition, it is worth noting that TRPV4 and rhodopsin could have a common pathway via which light would affect sperm. At this respect, the transduction pathways triggered by both TRPV4 and rhodopsin are related to the Ca^2+^ and calmodulin-dependent protein kinase C (PKC; see Kelleher and Johnson, 1986; Newton and Williams, 1991; Fan et al., 2009; Mundt et al., 2018). Because sperm mitochondrion plays a crucial role in calcium homeostasis (Rossi et al., 2019), the impact of light on TRPV4 and rhodopsin could ultimately affect that organelle.

In the light of all the aforementioned, this work sought to determine to which extent the mitochondrial electron-chain and the transduction pathways involving PKC, such as those activated by TRPV4 and rhodopsin, are involved in the sperm response to red light. For this purpose, pig sperm were irradiated with red LED light for 1, 5 or 10 min in the absence or presence of antimycin A, a specific inhibitor of the cytochrome C reductase activity and thus mitochondrial electron chain (Alexandre and Lehninger, 1984), and/or PKC 20–28^®^, a specific blocker of PKC. Following this, plasma membrane and acrosome integrity, motility, mitochondrial function (JC-1), O_2_ consumption, CCO activity, and levels of ROS, calcium and ATP were evaluated.

## Materials and methods

### Animals and semen samples

Sixteen semen samples coming from separate healthy Piétrain boars (2–3 years old) were provided by a commercial farm (Servicios Genéticos Porcinos, S.L. Roda de Ter, Spain). Animals were housed under climate-controlled conditions, fed with a standard diet and provided with water *ad libitum*. Ejaculates were collected through the gloved-hand method by specialized staff from the farm. The obtained sperm-rich fractions were immediately diluted at 17°C to a final concentration of 2×10^7^ sperm/mL in a commercial extender (Duragen^®^, Magapor, S.L.; Ejea de los Caballeros, Zaragoza, Spain). Diluted samples were then split into 90-ml artificial insemination doses; two of these doses were transported at 17°C to our laboratory. Transport of samples from the commercial farm to our laboratory lasted as much as 60 min and experimental procedures started upon arrival.

### Experimental design

All experiments were performed using eight independent biological samples, each resulting from two separate seminal doses. These samples came from eight different boars, all from the Pietrain breed. Samples were first checked to fulfil the following quality standards: ≥80% membrane-intact spermatozoa (SYBR14^+^/PI^−^), ≥85% morphologically normal spermatozoa, and ≥80% total motile spermatozoa. Thereafter, samples were split into four separate 1.5-ml aliquots and were either incubated for 10 min at 20°C in the dark (Control) or irradiated with red LED light at a wavelength range of 620–630 nm (PhastBlue^®^, IUL, S.L.; Barcelona, Spain) for 1 min (1′), 5 min (5′) or 10 min (10’). In all cases, the temperature within the PhastBlue^®^ system was maintained at 20°C. In addition to the aforementioned, samples were exposed to the same four protocols (i.e., control, or irradiation for 1, 5 or 10 min) in the presence of 100 nM of PKC 20–28^®^ (PKCi; Sigma Aldrich; St. Louis, MO, United States, catalog number 476480), 1 µM antimycin A (Sigma Aldrich, catalog number A8674), or both inhibitors together. Following this, motility; plasma membrane and acrosome integrity; mitochondrial membrane potential; intracellular levels of ROS, calcium and ATP; O_2_ consumption rate; and activities of PKC and cytochrome C oxidase (CCO) were determined.

In the case of intracellular ATP levels and activities of PKC and CCO, samples were first centrifuged at 2,000×g and 20°C for 30 s and the resulting pellet was frozen in liquid N_2_. Subsequently, samples were stored at -80°C until use, which did not last more than 1 month. For the evaluation of O_2_ consumption, samples were also incubated with either 5 µM oligomycin A, a specific inhibitor of ATP synthase activity (Grüber et al., 1994), or 5 µM carbonyl cyanide-4 (trifluoromethoxy) phenylhydrazone (FCCP), an uncoupling agent of the electron chain (Grasmick et al., 2018), alone (control) or together with PKCi and/or antimycin A. The use of oligomycin A and FCCP aimed to gain further insights into how light stimulation affects O_2_ consumption rate.

### Determination of protein kinase C activity

A first step was to determine total PKC activity to ascertain the ability of PKCi to induce its inhibitory effect. The description of this technique is provided in Supplementary File S1.

### Evaluation of sperm motility

Because motility is one of the most relevant features of sperm, a careful analysis of this parameter was needed. As in the case of PKC activity, the description of this technique is shown in Supplementary File S1.

### Flow cytometry

Flow cytometry analysis was performed using a CytoFLEX cytometer (Beckman Coulter, California, United States). Sperm viability (SYBR-14/PI), mitochondrial membrane potential (JC-1), total intracellular ROS levels (H_2_DCFDA/PI), superoxides (HE/YO-PRO-1), acrosome membrane integrity (PNA-FITC/PI) and intracellular calcium levels (Fluo3-AM/PI) were evaluated. In all cases, sperm were previously diluted with pre-warmed PBS to a final concentration of 1×10^6^ sperm per mL before they were stained with the corresponding protocol. SYBR-14, Fluo3-AM, YO-PRO-1, PNA-FITC, H2DCFDA and JC-1 monomers were excited with the 488 nm laser and their fluorescence was detected by the FITC channel (525/40). HE and JC-1 aggregates were excited with the 488 nm laser and its fluorescence was detected through the PE channel (585/42). PI was excited with the 488 nm laser and its fluorescence was detected by the PC5.5 channel (690/50).

Sperm viability was determined by analyzing plasma membrane integrity following incubation with SYBR-14 (final concentration: 31.8 nM) and PI (final concentration: 7.6 µM) at 38°C for 10 min, as described by Garner and Johnson (1995). Sperm with an intact plasma membrane (SYBR14^+^/PI^−^) were distinguished from those showing different degrees of plasma membrane alterations (SYBR14^-^/PI^+^ and SYBR14^+^/PI^+^).

Mitochondrial membrane potential was evaluated following a modified protocol from Ortega-Ferrusola et al. (2007). All samples were incubated with JC-1 (final concentration: 750 nM) at 38°C in the dark for 30 min. High mitochondrial membrane potential leads to the formation of JC-1 aggregates that emit orange fluorescence, whereas low mitochondrial membrane potential keeps JC-1 as monomers that emit green fluorescence.

Total ROS levels were determined through co-staining with H_2_DCFDA (final concentration: 100 µM) and PI (final concentration: 6 µM) at 38°C for 20 min, as described by Guthrie and Welch (2006). The percentage and geometric mean of fluorescence intensity of DCF^+^ in the subpopulation of viable sperm with high ROS levels (DCF^+^/PI^−^) were recorded.

The assessment of intracellular superoxide levels was performed by co-staining with HE (final concentration: 5 µM) and YO-PRO-1 (final concentration: 31.25 nM) at 38°C in the dark for 20 min, following a modification of the protocol of Guthrie and Welch (2006). The percentage and geometric mean of fluorescence intensity of E^+^ in the subpopulation of viable sperm with high superoxide levels (E^+^/YO-PRO-1^-^) were recorded.

Acrosome membrane integrity was evaluated following a procedure modified from Nagy et al. (2003). Sperm samples were incubated with PNA-FITC (final concentration: 1.17 µM) and PI (final concentration: 5.6 µM) at 38°C in the dark for 10 min. This procedure allowed the identification of four subpopulations. One of these subpopulations corresponded to viable sperm with an intact acrosome membrane (PNA-FITC^-^/PI^−^), and the other three comprised those sperm cells that had different degrees of alteration in plasma membrane and acrosome membranes (PNA-FITC^+^/PI^−^, PNA-FITC^-^/PI^+^ and PNA-FITC^+^/PI^+^).

Intracellular calcium levels of sperm were determined following the protocol described by Harrison et al. (1996) as modified by Yeste et al. (2015). Samples were incubated 10 min at 38°C with Fluo3-AM (final concentration: 1.2 µM) and PI (final concentration: 5.6 µM) at 38°C in the dark for 10 min. Four sperm subpopulations were identified: 1) viable sperm with low calcium levels (Fluo3^-^/PI^−^); 2) viable sperm with high calcium levels (Fluo3^+^/PI^−^); 3) non-viable sperm with low calcium levels (Fluo3^-^/PI^+^); and 4) non-viable sperm with high calcium levels (Fluo3^+^/PI^+^).

### Evaluation of mitochondrial membrane potential by confocal microscopy

Not only were sperm stained with JC-1 analyzed through flow cytometry, but they were also observed under a Leica TCS 4D confocal laser scanning microscope (Leica Lasertechnik; Wertrieb, Germany) adapted to an inverted Leitz DMIRBE microscope at 63× (NA = 1.4 oil) Leitz Plan-Apo Lens (Leitz; Stuttgarts, Germany) in which the light source was an argon/krypton laser (74 mW). Observations were carried out in 4-well plates, each well containing 20 µL of sample. Fluorescence was excited at 488 nm and detected at 530 nm for green emission and at 590 nm for the orange/red one. In addition, motility of stained sperm was recorded through sequential tracking at a velocity of one capture/s for 20 s (Blanco-Prieto et al., 2020). All biological replicates (n = 8) and treatments were examined, with a minimum of 200 sperm per sample.

### Determination of cytochrome C oxidase (CCO) activity

Activity of COO was analyzed in mitochondria-enriched sperm fractions, as described in Mclean et al. (1993). For this purpose, frozen pellets were solubilized in 500 µl of ice-cold PBS and then sonicated under the same conditions as those described for PKC activity. Thereafter, 500 µl of a 1.055 mg/ml Percoll^®^ solution in PBS at 4°C was placed onto each homogenate. Samples were subsequently centrifuged at 3,000×g and 10°C for 45 min. As a result, a mitochondria-enriched fraction appeared as a tight cloudy band that separated the aqueous phase from the Percoll^®^-enriched one. This band was carefully collected with a micropipette and transferred into 1.5-ml tubes, which were centrifuged at 12,000×g and 20°C for 2 min. As a final step, resulting pellets were resuspended in 100 µL PBS at 20°C. Mitochondria-enriched suspensions were finally split into two separate aliquots. The first aliquot was used to determine CCO activity through spectrophotometry with a commercial kit (Cytochrome C Oxidase Assay Kit; Sigma-Aldrich; catalogue number CYTOCOX1). The other aliquot, which had a volume of 10 μL, was used to determine the total protein content through a commercial kit based on the Bradford method (Bradford, 1976), as described above. Enzyme activity in each sample was calculated after normalizing the obtained spectrophotometric values against the total protein content.

### Determination of ATP levels

For determination of ATP levels, pellets prepared as described previously were thawed and resuspended in 300 µL of ice-cold 10 mM 2-[4-(2-hydroxyethyl)piperazin-1-yl]ethanesulfonic acid (HEPES) buffer containing 250 mM sucrose (pH 7.4), following the protocol set by Chida et al. (2012). Solubilized pellets were then sonicated with a Bandelin Sonopuls HD 2070 system (Bandelin Electronic GmbH and Co.) at a frequency of 10 kHz (20 pulses); samples were kept on ice at 4°C to avoid specimen heating. Following this, samples were centrifuged at 1,000×g and 4°C for 10 min and supernatants were collected. Twenty µl of each supernatant were used to determine the total protein content through the Bradford method (Bradford, 1976), using a commercial kit (Bio-Rad laboratories). The remaining volume was thoroughly mixed with 300 µl of ice-cold 10% (v:v) trichloroacetic acid, and then incubated at 4°C for 20 s. Subsequently, samples were centrifuged at 1,000×g and 4°C for 30 s, and the resulting supernatants were carefully separated from the pellet. These supernatants were again centrifuged at 1,000×g and 4°C for 10 min, obtaining a small, white pellet. The pellet was discarded, and the supernatant was mixed with two volumes of 1 M Tris-acetate buffer (pH = 7.75). Concentration of ATP in these resulting supernatants was determined through an Invitrogen^®^ ATP Determination Kit (ThermoFisher Bioscientific; Waltham, United States; catalogue number: A22066), following the manufacturer’s instructions. Samples were transferred into a 96-well microplate for fluorescence-based assays (Invitrogen^®^), and ATP levels were measured in an Infinite F200 fluorimeter (TECAN^®^; Männedorf, Switzerland). Data were normalized against the total protein content determined by the Bradford method.

### Determination of O_2_ consumption rate

The O_2_ consumption rate was determined with a SensorDish^®^ Reader (SDR) system (PreSens Gmbh; Regensburg, Germany), as described in Blanco-Prieto et al. (2020). For this purpose, 1-ml aliquots were transferred into a specifically-designed Oxodish^®^ OD24 plates (24 wells/plate). Plates were sealed with Parafilm^®^ and then placed into the SDR device, which was introduced in an incubator at 38°C. The O_2_ consumption was evaluated once a min for 2 h and, when finished, data were exported to an Excel file. The O_2_ consumption rate was calculated as the decrease of O_2_ concentration in each well during the 2-h incubation period, normalized against the percentage of viable sperm (SYBR14^+^/PI^−^) determined by flow cytometry.

As mentioned in the Experimental design section and to delve further into how irradiation with red light in the presence of antimycin A and/or PKCi affects O_2_ consumption, sperm were also incubated with 5 µM oligomycin A, a specific ATPase inhibitor, or 5 µM FCCP, a specific disruptor of the electron chain, as described in Blanco-Prieto et al. (2020). The inclusion of these additional samples allowed us to determine the following parameters (Supplementary Figure S1): 1) non-mitochondrial O_2_ consumption (NMC), which corresponded to O_2_ consumption recorded in the presence of 1 µM antimycin A; 2) basal respiratory rates (BR), which were the values obtained in the absence of any inhibitor/disruptor; 3) oligomycin-resistant O_2_ consumption (OC), which corresponded to samples incubated with 5 µM oligomycin A; 4) Maximal respiration (MR), which corresponded to samples incubated with 5 µM FCCP; 5) H^+^ leakage (HL): OC-NMC; 6) ATP turnover (AT): BR-HL; and 7) Spare respiratory capacity (SRC): MR-BR. Total O_2_ consumption rate resulted from the sum of NMC, BR and SRC, and, for all experimental conditions, the percentage of each of these components was calculated.

### Statistical analyses

Statistical analyses were conducted with a statistical package (SPSS^®^ Ver. 25.0 for Windows; IBM corp., Armonk, NY, United States). The first step was to test normality and homogeneity of variances through Shapiro-Wilk and Levene tests, respectively. When required, data were linearly transformed with √x or arcsin √x. The effects of light-stimulation and the presence/absence of antimycin A and PKCi on all variables (motility, plasma membrane and acrosome integrity, MMP, calcium levels, total ROS, superoxides, activities of PKC and CCO, ATP, and O_2_ consumption rate, including NMC, BR and SRC) were determined through a two-way analysis of variance (ANOVA) followed by post-hoc Sidak test. In this model, factors were: 1) light-stimulation pattern (i.e. non-irradiation, irradiation for 1 min, irradiation for 5 min, and irradiation for 10 min); and 2) presence/absence of inhibitors (control, antimycin A and PKCi, as well as oligomycin A and FCCP in the case of O_2_ consumption rate analysis).

On the other hand, motile sperm subpopulations were also determined as described in Luna et al. (2017). Kinematic parameters corresponding to each sperm cell (VCL, VSL, VAP, BCF, ALH, DNC, absMAD and algMAD) were used to run a Principal Component Analysis (PCA). The obtained matrix was rotated through the Varimax method and normalized with the Kaiser approach. The regression scores assigned to each sperm cell for the new PCA components were used to run a two-step cluster analysis (log-likelihood distance; Schwarz’s Bayesian Criterion). Three motile sperm subpopulations were identified (SP1, SP2 or SP3) and the proportions of each of these subpopulations were used to run a two-way ANOVA and post-hoc Sidak test.

In all analyses, the level of significance was set at *p* ≤ 0.05.

## Results

### Total PKC activity

Neither light-stimulation, regardless of the pattern, nor the presence of antimycin A modified PKC activity. More details are provided in Supplementary File S1.

### Sperm motility

**FIGURE 1:**
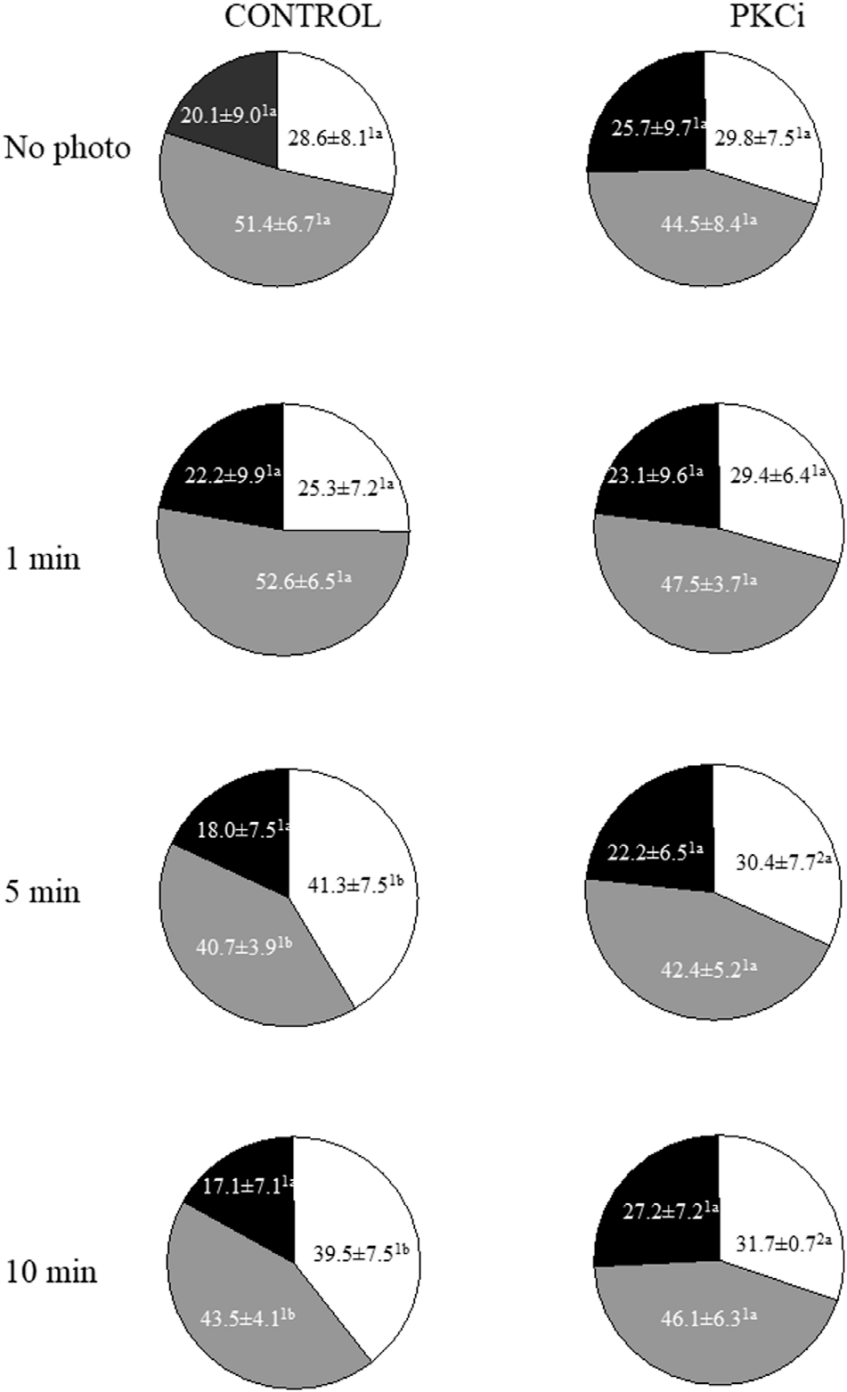
Proportions of motile sperm subpopulations following incubation with or without PKCi and further irradiation of sperm with red LED light. Sperm were not irradiated (No Photo) or irradiated for 1 min (1 min), 5 min (5 min) or 10 min (10 min) in the absence (CONTROL) or presence of 100 nM PKCi (PKCi). White sections: percentages of sperm included in Subpopulation 1. Grey sections: percentages of sperm included in Subpopulation 2. Black sections: percentages of sperm included in Subpopulation 3. Total number of analyzed sperm was 10,237. Different superscript letters indicate significant (*p* < 0.05) differences between samples that were light-irradiated with different patterns (i.e., 1, 5 or 10 min) and those that were not irradiated (i.e., 0 min), regardless of whether they were previously incubated with PKCi. Different superscript numbers indicate significant (*p* < 0.05) differences between samples incubated with PKCi and those non-incubated with the inhibitor, and irradiated with the same pattern (i.e., 1, 5 or 10 min). Eight biological replicates involving eight independent samples were examined.

Total and progressive sperm motility were not affected by irradiation with red light; these results are further described in Supplementary File S1. Regarding the structure of motile sperm subpopulations, three sperm subpopulations (SP) were identified, and their descriptive parameters are shown in Supplementary Table S2. These SPs were named in a descending order according to their VCL values. Proportions of SP1 (the fastest, based on VCL), SP2 and SP3 (the slowest) in non-irradiated samples without any inhibitor were 28.6% ± 8.1%, 51.4% ± 6.7% and 20.1% ± 8.1%, respectively (Figure 1). While these proportions remained unaffected when samples were irradiated for 1 min, those of SP1 increased and those of SP2 decreased when they were exposed to red light for 5 min or 10 min (*p* < 0.05; Figure 1). Remarkably, pre-incubation of sperm with PKCi abolished the aforementioned increase in the proportions of SP1 observed when samples were irradiated for 5 min or 10 min (Figure 1).

### Plasma membrane and acrosome integrity

Irradiation of sperm with red LED light did not affect plasma membrane or acrosome integrity. More information about the evaluation of these two sperm variables is provided in Supplementary File S1.

### Mitochondrial membrane potential

**FIGURE 2:**
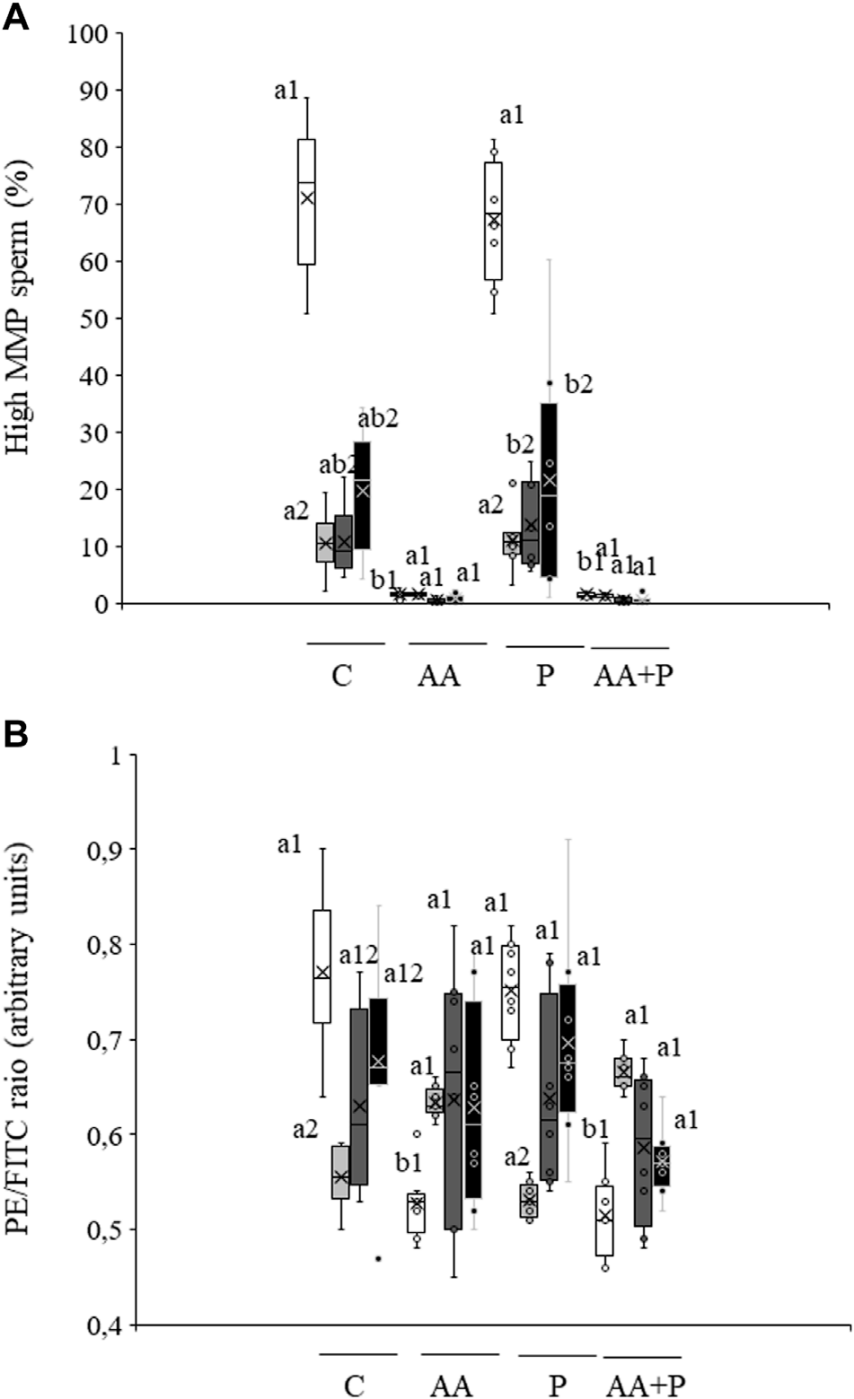
Box-and-whisker plot of the effects of antimycin A and PKCi on mitochondrial membrane potential. Sperm were subject to separate red LED light irradiation patterns in the presence of 1 µM antimycin A, 100 nM PKCi or both together, and parameters related to MMP were analyzed as described in Materials and Methods section. Sperm were not irradiated (white boxes) or irradiated with red LED light for 1 min (light grey boxes), 5 min (dark grey boxes) or 10 min (black boxes) in the absence (C) or presence of 1 µM antimycin A (AA), 100 nM PKCi (P) or 1 µM antimycin A and 100 nM PKCi together (AA + P). **(A)** percentages of sperm with high MMP. **(B)** Mean values of the JC-1_agg_/JC-1_mon_ ratio in the sperm population with high MMP. Figure shows means ± S.E.M. for eight biological replicates involving eight independent samples. Asterisks inside boxes indicate means, whereas lines inside represent medians. Different superscript numbers indicate significant (*p* < 0.05) differences between samples pre-incubated with the same inhibitor (i.e., Control, antimycin A, PKCi or antimycin A + PKCi) but subject to distinct irradiation patterns (i.e., 0, 1, 5 or 10 min). Different superscript letters indicate significant (*p* < 0.05) differences between samples incubated with separate inhibitors (Control, antimycin A, PKCi, or antimycin A + PKCi) and irradiated with the same pattern.

The percentage of sperm with high MMP, which in non-irradiated control samples was 71.0% ± 4.6%, did not vary when sperm were pre-incubated with PKCi (Figure 2A). Light irradiation induced a significant (P˂0.05) decrease in the percentage of sperm with high MMP at all tested irradiation times (Figure 2A). Preincubation with PKCi did not significantly modified the light-induced effect. Otherwise, pre-incubation with antimycin A, either alone or together with PKCi, led to an almost complete reduction of the percentage of sperm with high MMP in both control and irradiated samples, regardless of the irradiation time (Figure 2A).

In the absence of inhibitors, the ratio between JC-1 aggregates and JC-1 monomers (JC-1agg/JC-1mon) in the sperm population with high MMP significantly (*p* < 0.05) decreased after irradiation for 1 min (non-irradiated, control samples: 0.56 ± 0.01 vs. irradiation for 1 min: 0.77 ± 0.03; see Figure 2B). In both non-irradiated and irradiated samples, pre-incubation with antimycin A, either alone or together with PKCi, also induced a significant (*p* < 0.05) decrease in this ratio (Figure 2B). In contrast, pre-incubation with PKCi had no effect, either in non-irradiated or irradiated samples (Figure 2B).

When sperm stained with JC-1 were examined under a confocal laser scanning microscope, orange-stained mitochondria were mainly located at the two mid-piece poles in control samples and in those pre-incubated with PKCi (Supplementary Figure S4). Conversely, sperm pre-incubated with antimycin A did not show orange-stained mitochondria, but all were uniformly stained in green (Supplementary Figure S4). No irradiation pattern modified these specific distributions (data not shown).

### Intracellular calcium levels

As shown in Supplementary Figure S5, the percentage of viable sperm with high calcium levels was low in non-irradiated, control samples (0.55% ± 0.20%). Neither irradiation nor antimycin A or PKCi affected that percentage or the geometric mean intensity of Fluo3^+^ in the subpopulation of viable sperm with high calcium (Fluo3^+^/PI^−^; Supplementary Figure S5B).

### Total ROS

**FIGURE 3:**
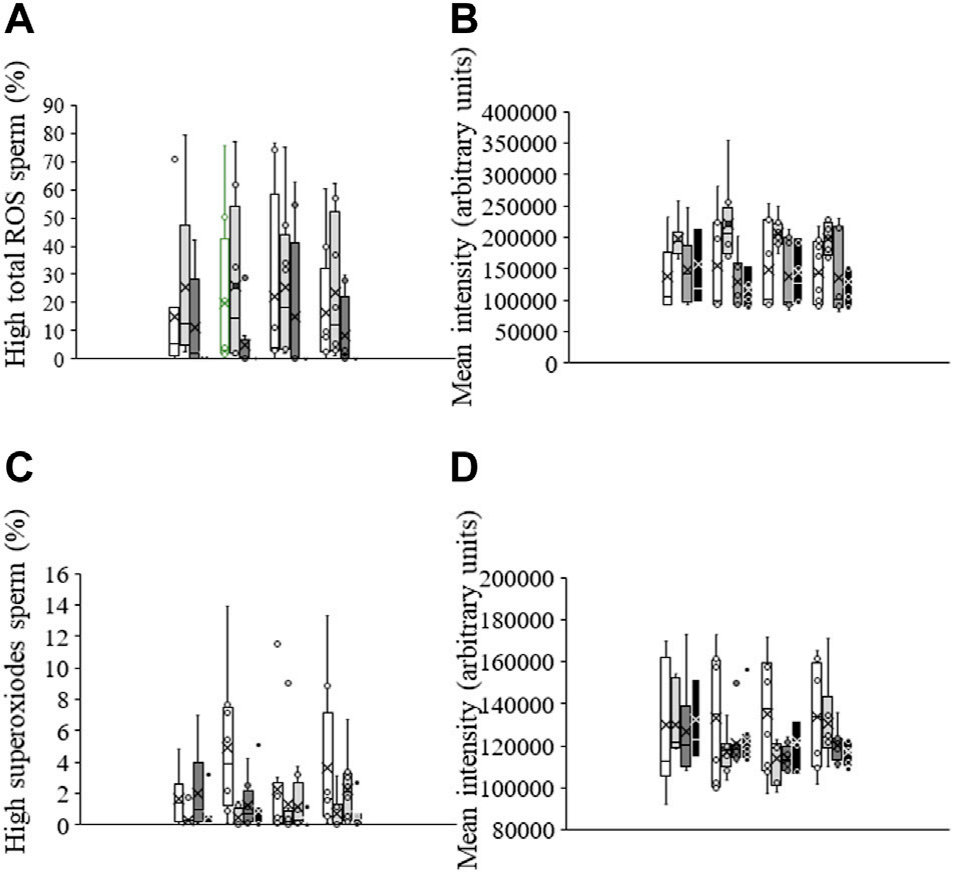
Box-and-whisker plot of the effects of antimycin A and PKCi on intracellular levels of total ROS and superoxides. Sperm were subject to separate red LED light irradiation patterns in the presence of 1 µM antimycin A, 100 nM PKCi or both together, and parameters related to ROS were analyzed as described in Materials and Methods section. Sperm were not irradiated (white boxes) or irradiated with red LED light for 1 min (light grey boxes), 5 min (dark grey boxes) or 10 min (black boxes) in the absence (C) or presence of 1 µM antimycin A (AA), 100 nM PKCi (P) or 1 µM antimycin A and 100 nM PKCi together (AA + P). **(A)** percentages of viable sperm with high ROS levels. **(B)** Fluorescence intensity of DCF^+^ in the population of viable sperm with high ROS levels (DCF^+^/PI^−^). **(C)** percentages of viable sperm with high superoxide levels. **(D)** Fluorescence intensity of E^+^ in the population of viable sperm with high superoxide levels (E^+^/YO-PRO-1^-^). Figure shows means ± S.E.M. for eight biological replicates involving eight independent samples. Asterisks inside boxes indicate means, whereas lines inside represent medians.

The percentage of viable sperm with high total ROS levels in non-irradiated, control samples was 15.1% ± 8.4% (DCF^+^/PI^−^; Figure 3A). No significant differences in this percentage or in the geometric fluorescence intensity of DCF^+^ in the subpopulation of viable sperm with high ROS were observed following irradiation or when samples were pre-incubated with PKCi. Sperm pre-incubated with antimycin A, however, and irradiated for 5 min or 10 min showed a significantly (*p* < 0.05) lower geometric mean fluorescence of DCF^+^-intensity than those irradiated for 1 min (Figure 3B).

### Superoxides

The percentage of viable sperm with high superoxide levels was very low in non-irradiated, control sperm, and lower than that of viable sperm with total ROS (1.6% ± 0.6%; Figure 3C). While irradiation did not induce a significant change in this parameter, percentages of viable sperm with high superoxide levels in samples pre-incubated with antimycin A were significantly (*p* < 0.05) lower after irradiation for 1 min or 10 min than when not exposed to light (Figure 3C). Finally, the geometric mean fluorescence of E^+^-intensity in the subpopulation of viable sperm with high superoxide levels (E^+^/YO-PRO-1^-^) was not affected by irradiation patterns or the presence of antimycin A or PKCi (Figure 3D).

### Cytochrome C oxidase activity and intracellular ATP levels

**FIGURE 4:**
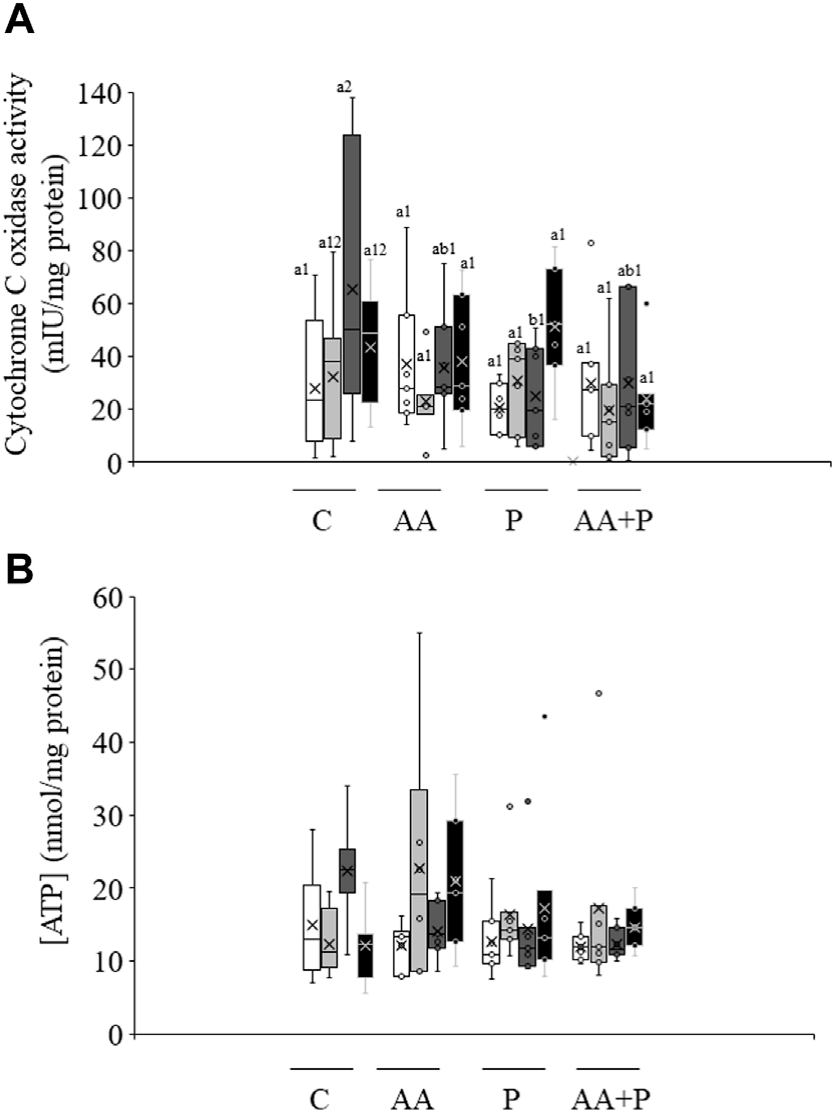
Box-and-whisker plot of the effects of antimycin A and PKCi on intracellular ATP levels and total cytochrome C oxidase activity. Sperm were subject to separate red LED light irradiation patterns in the presence of 1 µM antimycin A, 100 nM PKCi or both together, before intracellular ATP levels and cytochrome C oxidase activity were evaluated as described in Materials and Methods section. Sperm were not irradiated (white boxes) or irradiated with red LED light for 1 min (light grey boxes), 5 min (dark grey boxes) or 10 min (black boxes) in the absence (C) or presence of 1 µM antimycin A (AA), 100 nM PKCi (P) or 1 µM antimycin A and 100 nM PKCi together (AA + P). **(A)** intracellular ATP levels. **(B)** cytochrome C oxidase activity. Figure shows means ± S.E.M. for eight biological replicates involving eight independent samples. Asterisks inside boxes indicate means, whereas lines inside represent medians. In Figure 4A, different superscript numbers indicate significant (*p* < 0.05) differences between samples pre-incubated with the same inhibitor (i.e., Control, antimycin A, PKCi or antimycin A + PKCi) but subject to distinct irradiation patterns (i.e., 0, 1, 5 or 10 min). Different superscript letters indicate significant (*p* < 0.05) differences between samples incubated with separate inhibitors (Control, antimycin A, PKCi, or antimycin A + PKCi) and irradiated with the same pattern. In the case of Figure 4B, superscripts were not needed as no significant differences were found.

As shown in Figure 4A, irradiation of sperm for 5 min significantly (*p* < 0.05) increased CCO activity (irradiation for 5 min: 65.2 mIU/mg protein±19.1 mIU/mg protein vs. non-irradiated, control sperm: 28.1 mIU/mg protein±9.6 mIU/mg protein). Yet, this increase was abolished when samples were previously incubated with PKCi. Moreover, no changes in CCO activity were observed in samples pre-incubated with antimycin A. Otherwise, neither light-stimulation nor antimycin A or PKCi altered intracellular ATP levels, which in non-irradiated, control samples were 15.0 nmol/mg protein±2.7 (Figure 4B).

### O_2_ consumption rate

**FIGURE 5:**
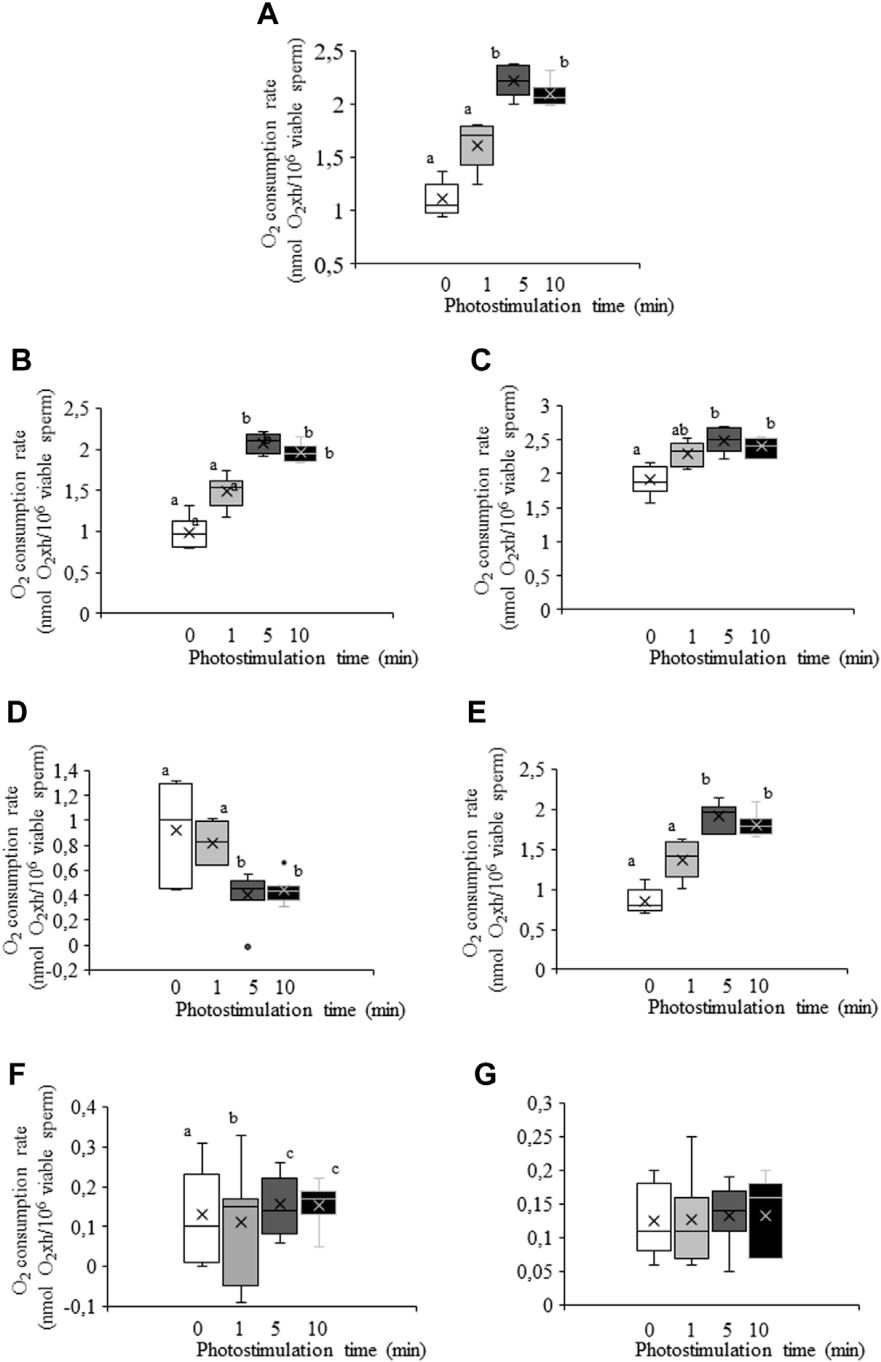
Box-and-whisker plot of the effects of red LED light irradiation on O_2_ consumption rate. Sperm were subject to separate red LED light irradiation patterns before total O_2_ consumption rate was analyzed and divided into its components as described in Materials and Methods section. Sperm were not irradiated (white boxes) or irradiated with red LED light for 1 min (light grey boxes), 5 min (dark grey boxes) or 10 min (black boxes). **(A)** total consumption rate, defined as the sum of non-mitochondrial O_2_ consumption, basal respiratory rate and spare respiratory capacity. **(B)** basal respiration. **(C)** maximal respiration. **(D)** spare respiratory capacity. **(E)** ATP turnover. **(F)** H^+^ leak. **(G)** non-mitochondrial O_2_ consumption. Figure shows means ± S.E.M. for 8 separate experiments. Figure shows means ± S.E.M. for eight biological replicates involving eight independent samples. Asterisks inside boxes indicate means, whereas lines inside represent medians. Different superscript letters indicate significant (*p* < 0.05) differences between irradiation patterns (i.e., 0, 1, 5 or 10 min). Superscripts were not needed in Figure 5G, as no significant differences were found.

**FIGURE 6:**
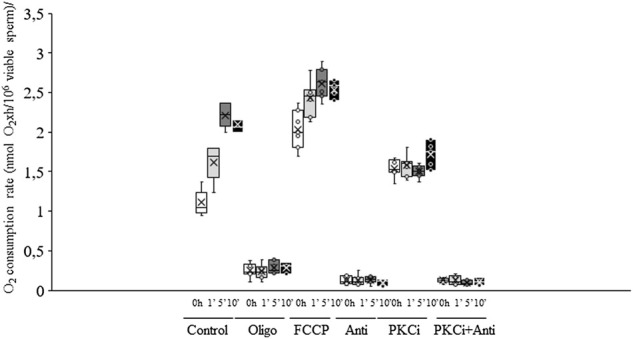
Box-and-whisker plot of the effects of antimycin A, PKCi, oligomycin A and FCCP on total O2 consumption rate. Sperm were subject to separate red LED light irradiation patterns in the presence of 1 µM antimycin A, 100 nM PKCi, 1 µM antimycin A+100 nM PKCi, 5 µM oligomycin A or 5 µM FCCP. Total O2 consumption rate, defined as the sum of non-mitochondrial O2 consumption, basal respiratory rate and spare respiratory capacity, was analyzed as described in Materials and Methods section. Sperm were not irradiated (white boxes) or irradiated with red LED light for 1 min (light grey boxes), 5 min (dark grey boxes) or 10 min (black boxes) in the absence (C) or presence of 1 µM antimycin A (AA), 100 nM PKCi (P) or 1 µM antimycin A+100 nM PKCi (AA + P). Figure shows means ± S.E.M. for eight biological replicates involving eight independent samples. Asterisks inside boxes indicate means, whereas lines inside represent medians. Different superscript numbers indicate significant (p < 0.05) differences between samples pre-incubated with the same inhibitor (i.e., Control, antimycin A, PKCi or antimycin A + PKCi) but exposed to distinct irradiation patterns (i.e., 0, 1, 5 or 10 min). Different superscript letters indicate significant (p < 0.05) differences between samples incubated with separate inhibitors (Control, antimycin A, PKCi, antimycin A + PKCi, oligomycin A or FCCP) and irradiated with the same pattern. Incubation with oligomycin A and FCCP was needed to determine basal respiration, maximal respiration, spare respiratory capacity, ATP turnover, H+ leak and non-mitochondrial O2 consumption.

Irradiation of sperm for either 5 min or 10 min induced a significant (*p* < 0.05) increase in total O_2_ consumption rate (non-irradiated, control samples. 1.11 nmol O_2_×h/10^6^ viable sperm±0.06 nmol O_2_×h/10^6^ viable sperm vs. irradiation for 10 min: 2.09 nmol O_2_×h/10^6^ viable sperm±0.04 nmol O_2_×h/10^6^ viable sperm; Figure 5A, Figure 6). This increase in total O_2_ consumption rate was based upon significant (*p* < 0.05) increases in basal and maximal levels of respiration, and ATP turnover rate, which were concomitant with a significant (*p* < 0.05) decrease in the reserve capacity (Figures 5B–D). In contrast, light-stimulation did not affect H^+^ leakage or non-mitochondrial O_2_ consumption (Figure 5E). On the other hand, while pre-incubation with antimycin A, either alone or in combination with PKCi, almost suppressed mitochondrial O_2_ consumption, this inhibition was not counteracted by red light irradiation (Figure 6). Pre-incubation with PKCi significantly (*p* < 0.05) increased O_2_ consumption rate in non-irradiated samples (PKCi: 1.54 nmol O_2_×h/10^6^ viable sperm±0.04 nmol O_2_×h/10^6^ viable sperm vs. control: 1.11 nmol O_2_×h/10^6^ viable sperm±0.06 nmol O_2_×h/10^6^ viable sperm; Figure 6). Pre-incubation with PKCi, nevertheless, prevented the aforementioned increase in total O_2_ consumption observed after irradiation for 5 min or 10 min (Figure 6). This effect occurred together with a decrease in SRC without affecting AT (Supplementary Figure S6A). When sperm were pre-incubated with PKCi and were subsequently irradiated for 1 min or 5 min, a decrease in BR and an increase in SRC were observed (*p* < 0.05; Supplementary Figure S6B). These effects were less apparent when samples were irradiated for 10 min, as only the increase in SRC was significant (*p* < 0.05; Supplementary Figure S6A). Finally, neither PKCi nor antimycin A had an impact on NMC (Supplementary Figure S6C).

## Discussion

Our results support that, whereas the mitochondrial electron chain is instrumental in explaining the effects of red light effects on sperm, those related to PKC only have a minor modulating role (mainly restricted to motility and CCO activity). Related with this, it is worth mentioning that previous research already suggested that indirect inhibition of the mitochondrial electron chain through blocking of ATP synthase induces effects compatible to those observed herein (Blanco-Prieto et al., 2020; Catalán et al., 2020d). The cytochrome C complex appears to be the most important component of the electron chain to explain the sperm response to red light, as it can be excited at 610–630 nm and 660–680 nm (Hollis et al., 2003). This reactivity could explain the light-induced activation of CCO observed after 5 min of irradiation. Furthermore, the fact that the activity of CCO was significantly increased after 5 min, but not after 1 min and 10 min, would indicate that the specific amount of energy absorbed by the cytochrome C complex follows the Arndt-Schulz curve (Lubart et al., 2006).

It is worth mentioning that antimycin A, which was used to inhibit the mitochondrial electron chain, acts through binding the Qi site of the cytochrome C reductase (CCR), a component of the cytochrome C complex included in Complex III (Huang et al., 2005). Inhibition of CCR activity by antimycin A blocks the oxidation of ubiquinol into ubiquinone, which, in turn, stops the entire electron chain (Huang et al., 2005). The fact that antimycin A blocked CCR would agree with the absence of response to red light when samples were previously incubated with this inhibitor. Moreover, not only was the abolishment of sperm response to red light in the presence of antimycin A apparent in the analysis of CCO activity, but also on MMP and intracellular superoxide levels. Interestingly, superoxide levels were more affected than total ROS when sperm were pre-incubated antimycin A. Total ROS include other reactive species, such as hydrogen peroxide and hydroxyl radical. While superoxide anion is known to be generated in complexes I and III of the electron chain (Brand et al., 2004), hydrogen peroxide results from the conversion of the previously formed superoxides by the superoxide dismutase complex (Boveris and Cadenas, 2000). Therefore, while the generation of superoxides is directly linked to the activity of complex III, which is inhibited by antimycin A, total ROS levels are only related indirectly. Additionally, whereas the effect of antimycin A on the electron chain was expected, that of the PKC inhibitor was less clear. One could indicate, on this respect, that ROS are known to regulate PKC activity, which is in turn linked to the modulation of mitochondria-controlled functions as relevant as apoptosis (see Cosentino-Gomes, 2012 for review). In fact, a direct relationship between the activity of PKC, which is also present inside mitochondria, and mitochondrial ROS, which is a by-product of the electron chain, has been established (Pakrash Mandal et al., 2021). Yet, while the present study supports the existence of a link between PKC and the electron chain, the exact mechanism of such a connection remains unknown.

Samples pre-incubated with antimycin A and irradiated for 5 min or 10 min altered the integrity of plasma membrane, although no effects on that of the acrosome were observed. A potential explanation could be the accumulation of energy in sperm incubated for 5 min or 10 min when the cytochrome C complex was blocked by antimycin A. Because red light has been reported to increase the percentage of sperm that reach the capacitated status (Yeste et al., 2016), it is reasonable to suggest that this accumulation of energy could elicit some para-apoptotic signal/s that would ultimately cause these alterations in the plasma membrane. In fact, cytochrome C is released upon permeabilization of mitochondrial matrix in one of the earliest apoptotic-like changes (Martinou and Green, 2001; Waterhouse et al., 2002). Because sperm capacitation shares some molecular mechanisms with apoptosis (Aitken et al., 2015) and red light has been reported to affect the ability of sperm to reach the capacitated status, it could be that these changes in plasma membrane integrity would result from an activation of a para-apoptotic mechanism, which other authors have named ‘spermptosis’ (Ortega-Ferrusola et al., 2017).

Changes observed in sperm motility also deserve an explanation. The effects of irradiation with red light observed herein were similar to those reported previously (Yeste et al., 2016; Pezo et al., 2019; Blanco-Prieto et al., 2020) and, as expected, pre-incubation with antimycin A led to sperm immobilization. In spite of this, this immobilization was not linked to a remarkable drop in intracellular energy, as ATP levels were not significantly altered by antimycin A. These results were similar to those observed when sperm were incubated with oligomycin A, before and after inducing *in vitro* sperm capacitation (Ramió-Lluch et al., 2013; Blanco-Prieto et al., 2020), even though the extent of that immobilization was larger in the case of antimycin A than in that of oligomycin A. The fact that ATP levels were maintained even when electron chain (antimycin A) or ATP synthase (oligomycin A) were blocked is not surprising, because most of the ATP produced in non-capacitated pig sperm comes from glycolysis, and only 3% at the most is generated by oxidative phosphorylation (Marín et al., 2003). These data, therefore, support that the regulation of sperm motility by mitochondria is not entirely related to the ATP produced by oxidative phosphorylation. Rather, other factors such as cation trafficking and intracellular redox balance could be involved in the regulation of sperm motility. In fact, voltage-dependent Ca^2+^ channels, such as CatSper and mitochondrial VDAC2, are involved in the regulation of sperm motility (Ren et al., 2001). This is important because mitochondria are the most important storage place for Ca^2+^ in sperm (Costello et al., 2009). Hence, the achievement of hyperactivated motility during capacitation requires a proper flux of Ca^2+^ through CatSper that would increase mitochondria activity (Sun et al., 2017). Likewise, mitochondria voltage channel VDAC2 is involved in the achievement of *in vitro* capacitation of pig sperm, including the achievement of hyperactivated motility patterns (Martínez-Abad et al., 2017). In both cases, capacitation is accompanied with a hike of intra-mitochondrial Ca^2+^ levels (Martínez-Abad et al., 2017; Sun et al., 2017). This increase was not observed in our conditions, but this is reasonable considering that sperm were maintained in basal, non-capacitating conditions. The electron chain arrest induced by antimycin A, nonetheless, would cause a severe change in mitochondria membrane electric polarity, due to the lack of H^+^ flux (Alberts et al., 2002). This alteration would affect the activity of mitochondrial voltage channels, such as VDAC2, and, in turn, the sperm functional parameters that depend on proper cation trafficking, such as motility (Lishko et al., 2012), which would ultimately lead to immobility. Thus, while the overall intra-mitochondrial calcium levels would not be affected, calcium trafficking through mitochondrial membranes would be altered, thus affecting the potential needed for the maintenance of motility (Bulkeley et al., 2021).

According to our results, whereas PKCi did not affect sperm motility, CCO activity or O_2_ consumption in non-irradiated samples, it did counteract the increase observed in the irradiated ones. A strong relationship between PKC activity and sperm motility has previously been reported (Naor and Breitbart, 1997). In our experiment, however, PKC inhibition was not complete, which would suggest that, in basal conditions, sperm motility would only require low PKC activity. This would be in accordance with previous reports, where sperm motility was found to be maintained at low PKC activity (Naor and Breitbart, 1997). In this way, this basal PKC activity, which would partially remain in the presence of PKCi, would be enough to maintain the observed basal motion characteristics.

Analysis of O_2_ consumption rate in non-irradiated, control sperm indicated that this parameter can be much increased, as its rise after light-stimulation demonstrated. In this context, interrogating on the role of this increase with regard to the lifespan of pig sperm is relevant. Previous studies demonstrated that, while O_2_ consumption rate is low - even after achieving the capacitated status (Ramió-Lluch et al., 2013; Blanco-Prieto et al., 2020) -, a transient increase occurs upon triggering the acrosome reaction (Ramió-Lluch et al., 2013). This peak could explain the high SRC observed in pig sperm, which require a great spare capacity to cover the sudden need for energy linked to acrosome exocytosis, as indicated by the O_2_ consumption peak (Ramió-Lluch et al., 2013). This process could be different in species where oxidative phosphorylation provides more ATP than in the pig, such as mice or humans (Mukai and Okuno, 2004; Nascimento et al., 2008). Remarkably, pre-incubation of sperm with PKCi counteracted the increase in O_2_ consumption rate observed in irradiated samples. On this respect, it is important to highlight that PKC depends on mitochondrial-related proteins, such as the mitochondrial, ATP-sensitive K^+^ channel mitoKATP, involved in the regulation of MMP and redox homeostasis (Queliconi et al., 2011). Based on these findings and given that the response of sperm to irradiation mediated by TRPV4and rhodopsin is related to PKC, one could suggest that the inhibition of PKC could ultimately affect the mitochondrial activity via modulation of proteins such as mitoKATP.

In summary, our results indicated that the response of sperm to red light is largely reliant upon the mitochondrial electron chain, since the complete blockage of that chain by antimycin A prevented light action and was not countered by irradiation. This response would be related to a direct action of light on mitochondrial photosensitizers, especially those of the electron chain. Moreover, other indirect mechanisms related to PKC, like those of TRPV4 and rhodopsin, would be less relevant for the response of pig sperm to red light. Yet, and because the inhibition of PKC counteracted the changes in motile sperm subpopulations induced by irradiation, one could suggest that these PKC-related mechanisms could have an effect on specific sperm function variables, such as CCO activity and motility.

## Data Availability

The raw data supporting the conclusions of this article will be made available by the authors, without undue reservation.
