# Supplementary figures and images for: The effects of red LED light on pig sperm function rely upon mitochondrial electron chain activity rather than on a PKC-mediated mechanism
Source: Front Cell Dev Biol. 2022 Oct 7;10:930855. doi: 10.3389/fcell.2022.930855 (PMC9585505; doi:10.3389/fcell.2022.930855)

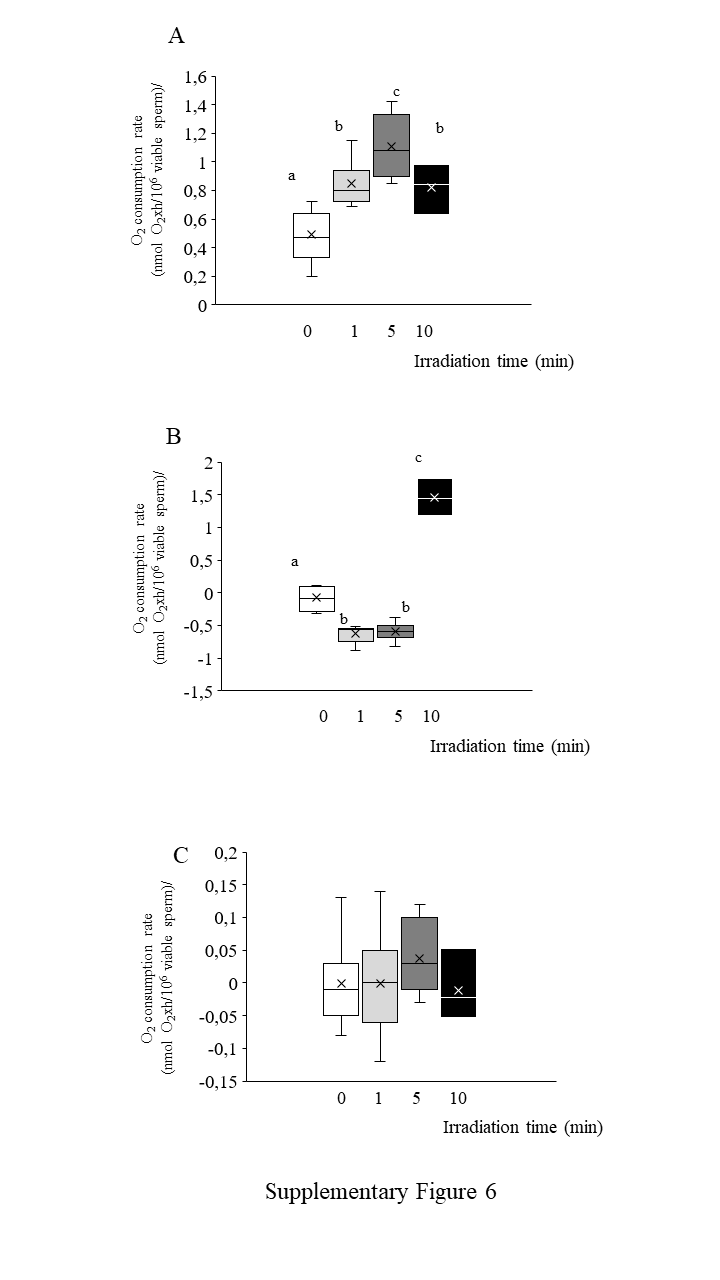

Supplement: Supplementary file 1 [file Image6.TIF]

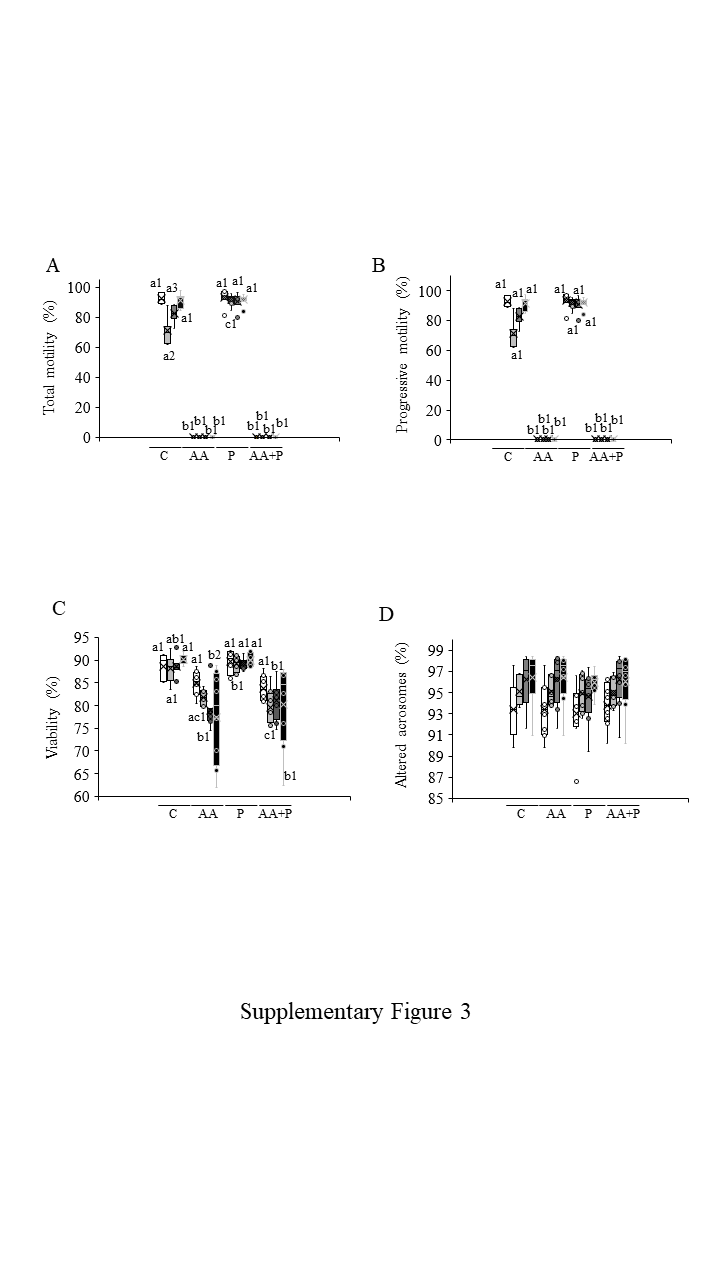

Supplement: Supplementary file 2 [file Image3.TIF]

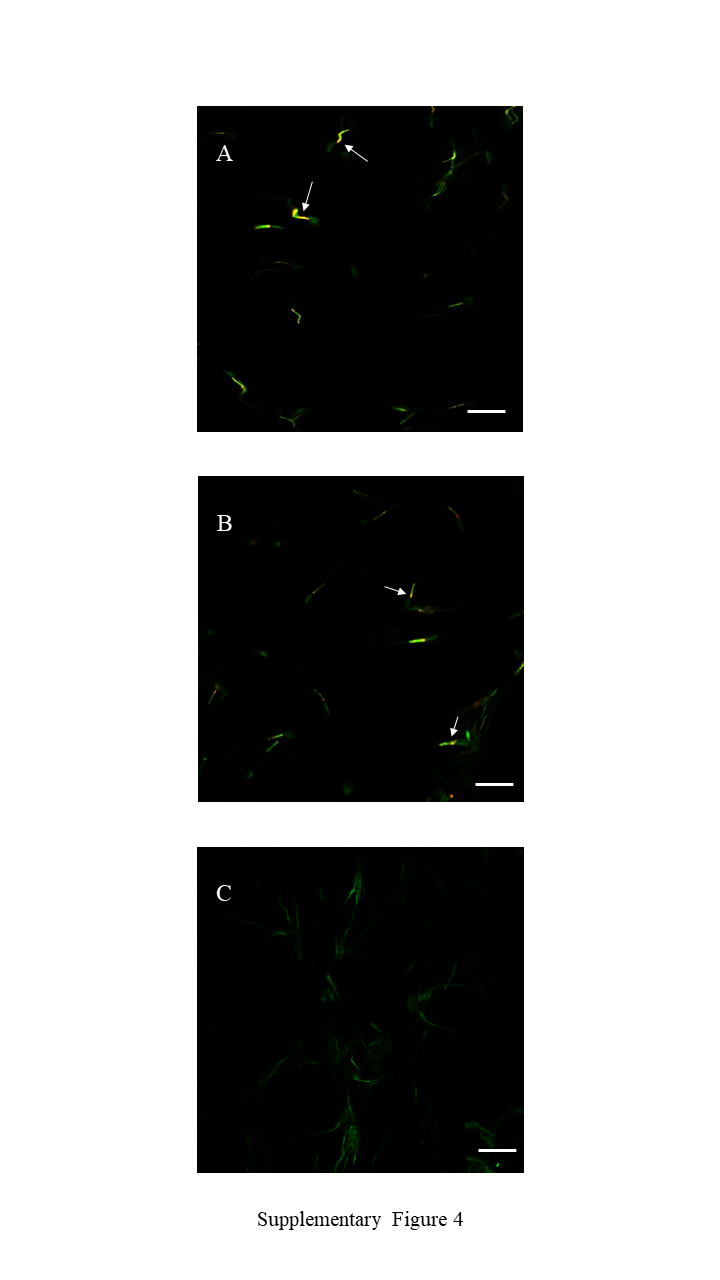

Supplement: Supplementary file 3 [file Image4.TIF]

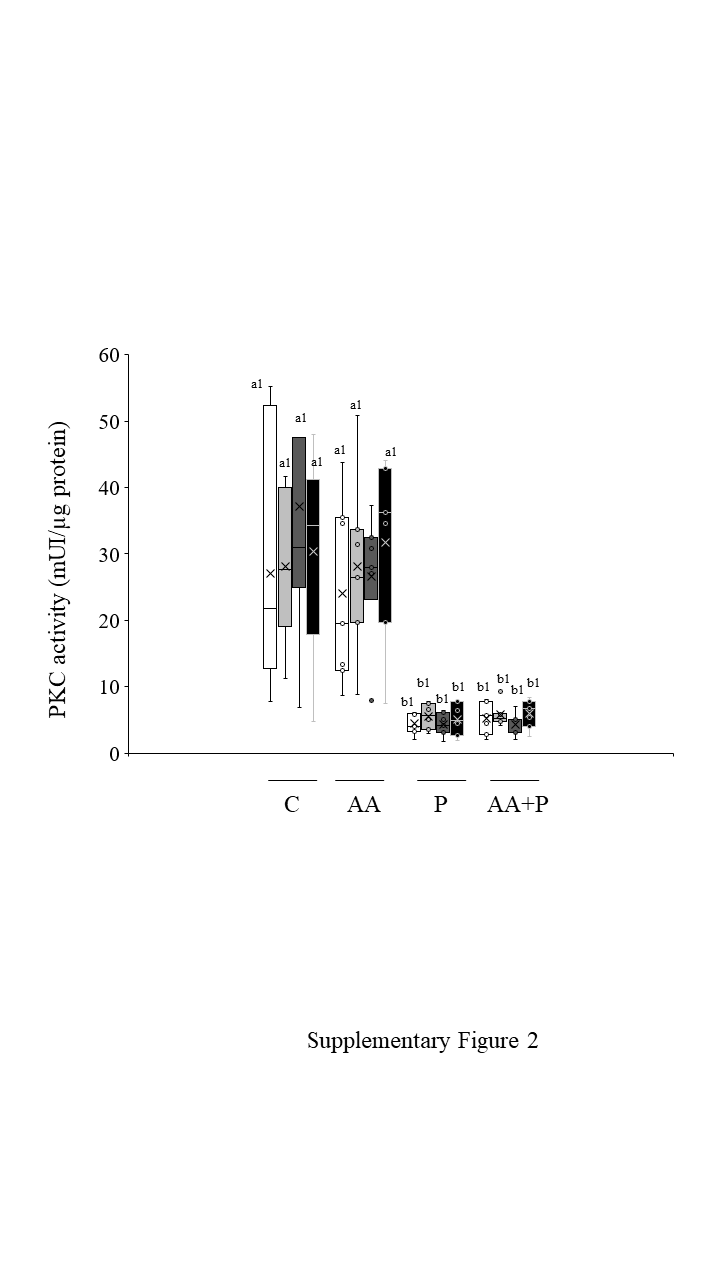

Supplement: Supplementary file 4 [file Image2.TIF]

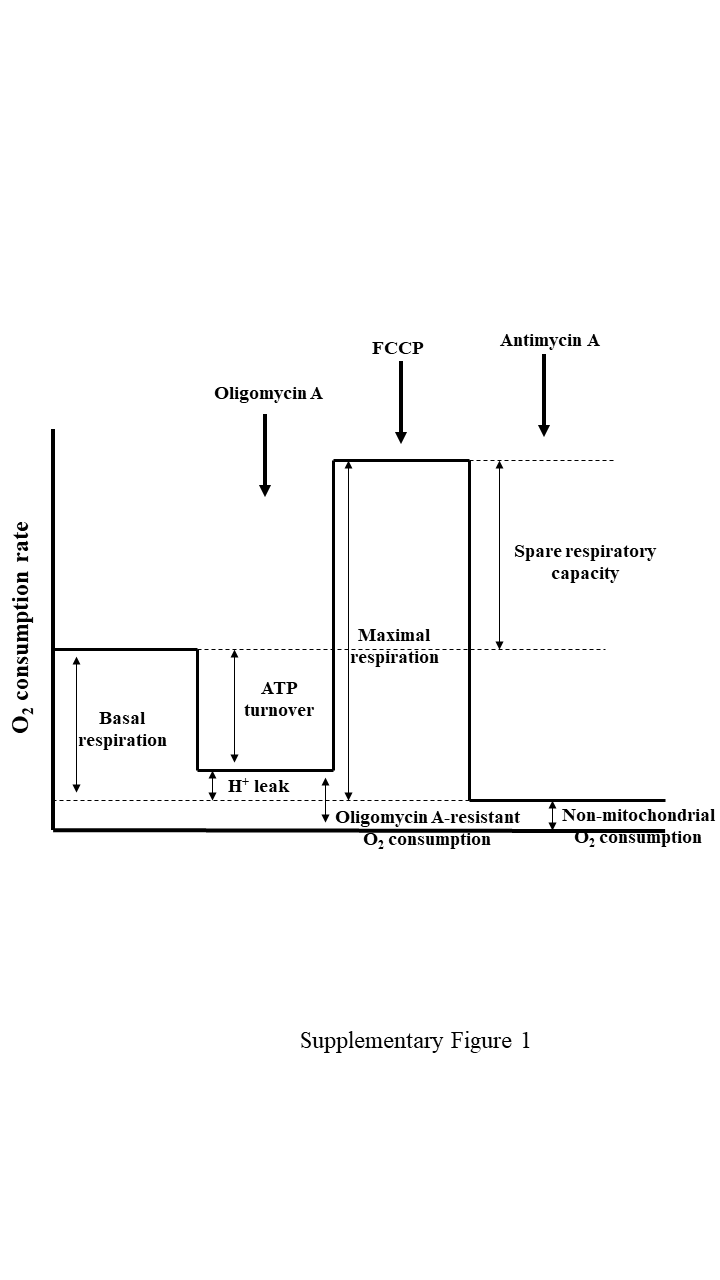

Supplement: Supplementary file 5 [file Image1.TIF]

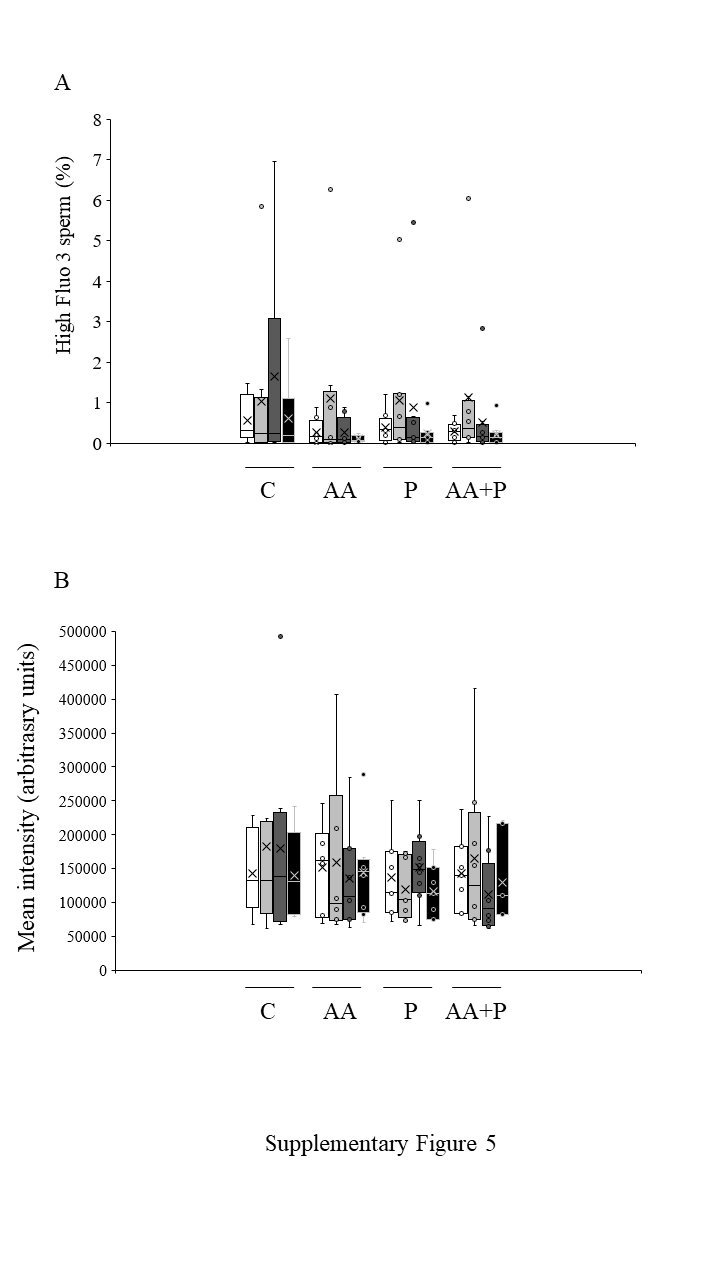

Supplement: Supplementary file 6 [file Image5.TIF]
