# Supplementary material for: The effects of red LED light on pig sperm function rely upon mitochondrial electron chain activity rather than on a PKC-mediated mechanism
Source: Front Cell Dev Biol. 2022 Oct 7;10:930855. doi: 10.3389/fcell.2022.930855 (PMC9585505; doi:10.3389/fcell.2022.930855)
Supplement: Supplementary file 7 [file DataSheet1.docx]

**Supplementary File 1**

**SUPPLEMENTARY MATERIALS AND METHODS**

## Determination of protein kinase C activity

Total PKC activity in sperm was determined following the procedure described by Rotem et al. (1990), using a commercial kit and following the manufacturer’s instructions (PKC Kinase Activity Assay Kit; Abcam; Cambridge, UK, catalog number 139437). Frozen pellets were thawed and diluted with 200 µL/sample of an ice-cold homogenization buffer (HB; pH 7.5) made up of 20 mM Tris/HCl, 250 mM sucrose, 10 mM ethylene glycol-bis(2-aminoethylether)-N,N,N’,N’,-tetraacetic acid (EGTA), 2 mM ethylene diamino tetraacetic acid (EDTA), 2 mM phenylmethyl sulfonil fluoride (PMSF) and 40 µg/mL leupeptin. Samples were subsequently sonicated using a Bandelin Sonopuls HD 2070 system (Bandelin Electronic GmbH and Co., Berlin, Germany) at a frequency of 10 kHz (20 pulses), keeping them on ice to avoid heating. Homogenized samples were then centrifuged at 12,000×g and 4 ºC for 60 min. The resulting supernatant was carefully collected and transferred into a separated tube, whereas the pellet was resuspended in 200 µL of an ice-cold HB supplemented with 0.5% (v:v) Triton X-100. Resuspended pellets were again homogenized by sonication under the aforementioned conditions. Samples were kept at 4 ºC for 30 min and then centrifuged at 12,000×g and 4 ºC for 30 min. Supernatants were collected and mixed with those obtained in the former step, and the resulting sample was used to evaluate PKC activity, and total protein content through the Bradford method (Bradford, 1976) and using a commercial kit (Bio-Rad laboratories; Hercules, Ca, USA). Finally, total PKC activity was normalized against the total protein of each sample.

## Evaluation of sperm motility

Sperm motility was analyzed using a CASA system (Integrated Sperm Analysis System V1.0; Proiser; Valencia, Spain), and the following parameters described in Blanco-Prieto et al. (2020) were recorded: curvilinear velocity (VCL), straight line velocity (VSL), average pathway velocity (VAP), linearity coefficient (LIN), straightness coefficient (STR), wobble coefficient (WOB), amplitude of lateral head displacement (ALH), beat cross frequency (BCF), dance (DNC, VCL×ALH), absolute mean angular displacement (absMAD) and algebraic mean angular displacement (algMAD). Before analysis, samples were warmed in a water bath at 38 ºC for 10 min. Afterwards, 6 µL of each sample were placed into a 20-micron Leja® Standard Count Chamber Slide (Leja Products B.V.; Nieuw Vennep, The Netherlands). Three replicates of at least 1,000 sperm each were evaluated, and the corresponding mean ± standard error of the mean (SEM) was calculated. Total motility was defined as the percentage of sperm with VAP≥10 µm/s, and progressive motility was defined as that of motile sperm with STR≥45%.

**SUPPLEMENTARY RESULTS**

**Total PKC activity**

Non-irradiated samples showed a PKC activity of 27.0 mIU/mg protein±6.7 mIU/mg protein (mean±S.E.M.; Suppl. Fig. 2). Neither light-stimulation, regardless of the pattern, nor the presence of antimycin A modified PKC activity. Incubation of sperm with PKCi, however, induced a significant (P<0.05) decrease in PKC activity, with values of 4.5 mIU/mg protein±0.5 mIU/mg protein in non-irradiated samples (Suppl. Fig. 2). This decrease was also observed in irradiated samples, with no differences between the irradiated and the non-irradiated. Activity of PKC when samples were incubated with both PKCi and antimycin A was similar to that of incubation with PKCi (Suppl. Fig. 2).

**Total motility, progressive motility and mean values of motility parameters**

Total sperm motility, which in non-irradiated control samples was 92.9%±1.3%, was not altered by light-stimulation (Suppl. Fig. 3A). Although total motility in sperm pre-incubated with PKCi significantly (P<0.05) increased following irradiation for 1 min (samples pre-incubated with PKCi and irradiated for 1 min: 91.8%±1.4% vs. samples not pre-incubated with PKCi and irradiated for 1 min: 71.1%±3.3%; Suppl. Fig. 3A), this effect was not observed when samples were irradiated for 5 min or 10 min. Moreover, pre-incubation with antimycin A or with antimycin A+PKCi induced a complete immobilization of sperm in both non-irradiated and irradiated samples (Suppl. Fig. 3A).

Evaluation of progressive motility showed similar results but, in this case, pre-incubation with PKCi had no effect on either non-irradiated or irradiated samples (Suppl. Fig. 3B). Because antimycin A completely inhibited motility, sperm did not exhibit progressive motility (Suppl. Fig. 3B).

As shown in Suppl. Table 1, kinematic parameters were affected by light-stimulation. In samples that were not pre-incubated with antimycin A or PKCi, irradiation for 10 min but not for 1 min or 5 min significantly (P<0.05) increased BCF (10 min: 8.57 Hz±0.28 Hz vs. non-irradiated samples: 7.64 Hz±0.21 Hz). In addition, BCF was significantly (P<0.05) higher in samples pre-incubated with PKCi and irradiated for 1 min and 5 min than in their counterparts not pre-incubated with that inhibitor. Moreover, pre-incubation with PKCi significantly (P<0.05) increased VCL in non-irradiated and irradiated sperm. A significant (P<0.05) increase in DNC was observed in samples pre-incubated with PKCi and irradiated for 5 min compared to those non-irradiated and not pre-incubated with PKCi (5 min: 242.4 µm^2^/s±2.8 µm^2^/s vs. non-irradiated: 221.6 µm^2^/s ±21.0 µm^2^/s).

**Plasma membrane and acrosome integrity**

The percentage of sperm with an intact plasma membrane (SYBR14^+^/PI^-^; viable sperm) in non-irradiated samples and without either antimycin A or PKCi was 88.7%±1.0% (Suppl. Fig. 3C); no irradiation pattern modified this percentage. In the case of samples pre-incubated with antimycin A, whereas no significant differences were observed between non-irradiated samples and those irradiated for 1 min, a significant (P<0.05) decrease in the percentage of sperm with an intact plasma membrane was observed when samples irradiated for 5 min and 10 min were compared with those irradiated with the same patterns in the absence of antimycin A (Suppl. Fig. 3C). Similar results were observed when samples were pre-incubated with both antimycin A and PKCi; in this case, significant differences were observed in all irradiation patterns (i.e., 1 min, 5 min and 10 min). In contrast, pre-incubation with PKCi alone had no effect (Suppl. Fig. 3C).

On the other hand, the percentage of viable sperm with an intact acrosome in non-irradiated, control samples was high (93.8%±3.3%; see Suppl. Fig. 3D). No significant differences between irradiated and non-irradiated samples were observed either in the presence or absence of antimycin A or PKCi (Suppl. Fig 3D).

**Supplementary Figure 1.** Diagram representing the different components related to O_2_ consumption: non-mitochondrial O_2_ consumption, basal respiratory rate, oligomycin-resistant O_2_ consumption, maximal respiration, H^+^ leak, ATP turnover and spare respiratory capacity.

**Supplementary Figure 2.** Box-and-whisker plot of the effects of irradiating sperm with red LED light, after incubation with or without PKCi or antimycin A on PKCi activity. Sperm were not irradiated (white boxes) or irradiated for 1 min (light grey boxes), 5 min (dark grey boxes) or 10 min (black boxes) in the absence (C) or presence of 1 µM antimycin A (AA), 100 nM PKCi (P) or 1 µM antimycin A plus 100 nM PKCi (AA+P). Asterisks inside boxes indicate means, whereas lines inside represent medians. Figure shows means±S.E.M. for eight biological replicates involving eight independent samples. Different superscript numbers indicate significant (P<0.05) differences between samples pre-incubated with the same inhibitor (i.e., Control, antimycin A, PKCi or antimycin A+PKCi) but subject to distinct irradiation patterns (i.e., 0 min, 1 min, 5 min or 10 min). Different superscript letters indicate significant (P<0.05) differences between samples incubated with separate inhibitors (Control, antimycin A, PKCi, or antimycin A+PKCi) and irradiated with the same pattern.

**Supplementary Figure 3.** Box-and-whisker plot of the effects of irradiating sperm with red LED light, after incubation with or without PKCi or antimycin A on total (A) and progressive motility (B), and plasma membrane **(C)** and acrosome **(D)** integrity. Sperm were not irradiated (white boxes) or irradiated for 1 min (light grey boxes), 5 min (dark grey boxes) or 10 min (black boxes) in the absence (C) or presence of 1 µM antimycin A (AA), 100 nM PKCi (P) or 1 µM antimycin A plus 100 nM PKCi (AA+P). Asterisks inside boxes indicate means, whereas lines inside represent medians. Figure shows means±S.E.M. for eight biological replicates involving eight independent samples. Different superscript numbers indicate significant (P<0.05) differences between samples pre-incubated with the same inhibitor (i.e., Control, antimycin A, PKCi or antimycin A+PKCi) but subject to distinct irradiation patterns (i.e., 0 min, 1 min, 5 min or 10 min). Different superscript letters indicate significant (P<0.05) differences between samples incubated with separate inhibitors (Control, antimycin A, PKCi, or antimycin A+PKCi) and irradiated with the same pattern. Superscripts were not needed in Supplementary Figure 3D, as no significant differences were found.

**Supplementary Figure 4.** Representative images showing high-MMP (orange-stained) and low-MMP mitochondria (green-stained) in sperm incubated in the presence or absence of antimycin A and PKCi. Sperm were incubated in the absence (A) or presence of either 100 nM PKCi (B) or 1µM antimycin A (C). Arrows indicate the localisation of high MMP mitochondria. Scale bar: 40 µm. In this experiment, eight biological replicates involving eight independent samples were evaluated (200 sperm per sample/treatment).

**Supplementary Figure 5.** Box-and-whiskers plot of the effects of antimycin A and PKCi on intracellular calcium levels. Sperm were exposed to separate red LED light irradiation patterns in the presence of 1 µM antimycin A, 100 nM PKCi or 1 µM antimycin A+100 nM PKCi, before evaluation with Fluo3 staining as described in Materials and Methods section. Sperm were not irradiated (white boxes) or irradiated with red LED light for 1 min (light grey boxes), 5 min (dark grey boxes) or 10 min (black boxes) in the absence (C) or presence of 1 µM antimycin A (AA), 100 nM PKCi (P) or 1 µM antimycin A+100 nM PKCi together (AA+P). A: Percentages of viable sperm with high calcium levels. B: Fluorescence intensity of Fluo3^+^ in the viable sperm population with high calcium levels. Figure shows means±S.E.M. for eight biological replicates involving eight independent samples. Asterisks inside boxes indicate means, whereas lines inside represent medians. No superscripts were needed, as no significant differences between experimental conditions were found.

**Supplementary Figure 6.** Box-and-whisker plot of the effects of PKCi on the components of O_2_ consumption rate. Sperm were exposed to separate red LED light irradiation patterns in the presence of 100 nM PKCi, The rates of spare respiratory capacity (A), basal respiration (B) and non-mitochondrial O_2_ consumption (C) were analyzed as described in Materials and Methods section. Sperm were not irradiated (white boxes) or irradiated with red LED light for 1 min (light grey boxes), 5 min (dark grey boxes) or 10 min (black boxes) in the absence or presence of 100 nM PKCi. Boxes show differences between samples incubated with or without 100 nM PKCi, and then irradiated. Figure shows means±S.E.M. for eight biological replicates involving eight independent samples. Asterisks inside boxes indicate means, whereas lines inside represent medians. Different superscript letters indicate significant (P<0.05) differences between irradiation patterns. Superscripts were not needed in Supplementary Figure 6C, as no significant differences were found.

**Supplementary Table 1**. Kinetic parameters (mean ± SEM) of sperm not pre-incubated (control) or pre-incubated with PKCi, and then irradiated for 1 min, 5 min or 10 min, or not irradiated.

|  | Control | | | | PKCi | | | |
| --- | --- | --- | --- | --- | --- | --- | --- | --- |
|  | Non-irradiated | 1 min | 5 min | 10 min | Non-irradiated | 1 min | 5 min | 10 min |
| VCL (µm/s) | 69.6±3.7^a1^ | 67.0±3.1^a1^ | 69.7±3.8^a1^ | 71.2±4.0^a1^ | 74.8±4.7^b1^ | 78.4±5.5^b1^ | 78.0±5.8^b1^ | 73.2±4.3^b1^ |
| VSL (µm/s) | 42.8±2.0 | 40.5±2.2 | 42.9±2.4 | 40.8±2.0 | 44.9±2.9 | 45.8±2.8 | 45.7±2.9 | 42.8±3.0 |
| VAP (µm/s) | 53.4±3.1 | 51.8±2.7 | 55.7±4.2 | 53.3±4.0 | 57.6±4.9 | 59.9±6.1 | 59.5±6.3 | 57.4±5.8 |
| LIN (%) | 57.9±5.4 | 63.3±6.7 | 64.2±6.9 | 60.2±6.3 | 62.5±6.8 | 63.4±6.8 | 61.7±5.9 | 60.2±6.0 |
| STR (%) | 72.7±7.1 | 77.8±7.9 | 77.3±7.9 | 76.5±7.4 | 78.0±8.4 | 79.3±8.4 | 76.7±7.6 | 73.6±7.7 |
| WOB (%) | 72.4±7.5 | 79.4±8.2 | 81.1±8.9 | 76.8±8.0 | 78.6±7.8 | 81.5±9.1 | 78.2±7.7 | 79.9±8.0 |
| BCF (Hz) | 7.64±0.21^a1^ | 7.63±0.20^a1^ | 7.59±0.20^a1^ | 8.57±0.28^a2^ | 8.51±0.30^b1^ | 8.81±0.37^b1^ | 8.30±0.22^b12^ | 8.05±0.30^a2^ |
| mALH (µm) | 2.39±0.19 | 2.59±0.23 | 2.49±0.21 | 2.51±0.26 | 2.54±0.28 | 2.57±0.29 | 2.61±0.29 | 2.75±0.34 |
| DNC (µm^2^/s) | 209.2±18.6^a1^ | 208.4±18.1^a1^ | 204.5±17.9^a1^ | 209.0±18.7^a1^ | 221.6±21.0^a1^ | 229.3±23.4^a12^ | 242.4±2.8^a2^ | 229.3±23.5^a12^ |
| absMAD (º) | 74.8±4.9 | 77.1±5.6 | 74.2±4.7 | 84.4±6.5 | 82.7±6.4 | 84.5±7.3 | 82.8±6.7 | 74.9±5.0 |
| algMAD (º) | 0.22±0.27 | 0.16±0.29 | 0.10±0.19 | -0.25±0.28 | -0.15±0.21 | 0.16±0.22 | 0.13±0.18 | -0.30±0.29 |

Different superscript numbers indicate significant (P<0.05) differences between samples pre-incubated with the same inhibitor (i.e., Control, antimycin A, PKCi or antimycin A+PKCi) but subject to distinct irradiation patterns (i.e., 0 min, 1 min, 5 min or 10 min). Different superscript letters indicate significant (P<0.05) differences between samples incubated with separate inhibitors (Control, antimycin A, PKCi, or antimycin A+PKCi) and irradiated with the same pattern.

**Supplementary Table 2.** Kinematic parameters (mean ± SEM) of the three sperm motile subpopulations identified in this study.

|  | Subpopulation 1 | Subpopulation 2 | Subpopulation 3 |
| --- | --- | --- | --- |
| VCL (µm/s) | 45.5±4.0 | 74.4±4.9 | 111.5±5.3 |
| VSL (µm/s) | 29.7±3.2 | 45.7±2.8 | 58.8±3.5 |
| VAP (µm/s) | 37.2±3.6 | 59.4±4.5 | 79.2±5.4 |
| BCF (Hz) | 8.33±0.30 | 8.21±0.3.1 | 7.69±0.30 |
| ALH (µm) | 1.55±0.16 | 2.50 ±0.39 | 4.20±0.54 |
| DNC(µm^2^s) | 74.6± 21.6 | 192.2±24.3 | 483.0±51.1 |
| absMAD (º) | 70.8±4.1 | 78.8±5.8 | 95.4±7.5 |
| algMAD (º) | -0.44±0.91 | 1.08±2.05 | -1.39±2.36 |

Subpopulations were numbered based on descending VCL values.
